# MicroRNA Expression Signatures in Clear Cell Renal Cell Carcinoma: High-Throughput Searching for Key miRNA Markers in Patients from the Volga-Ural Region of Eurasian Continent

**DOI:** 10.3390/ijms24086909

**Published:** 2023-04-07

**Authors:** Irina Gilyazova, Elizaveta Ivanova, Adel Izmailov, Ildar Sharifgaliev, Alexandra Karunas, Elena Pudova, Anastasiya Kobelyatskaya, Gulshat Gilyazova, Angelina Izmailova, Valentin Pavlov, Elza Khusnutdinova

**Affiliations:** 1 Institute of Biochemistry and Genetics-Subdivision of the Ufa Federal Research Centre of the Russian Academy of Sciences, 450054 Ufa, Russia; 2 Institute of Urology and Clinical Oncology, Bashkir State Medical University, 450008 Ufa, Russia; 3 Republican Clinical Oncological Dispensary, 450054 Ufa, Russia; 4 Engelhardt Institute of Molecular Biology, Russian Academy of Sciences, 119991 Moscow, Russia

**Keywords:** renal cell carcinoma, miRNA, gene expression

## Abstract

Clear cell renal cell carcinoma (ccRCC) is characterized by high molecular genetic heterogeneity, metastatic activity and unfavorable prognosis. MicroRNAs (miRNA) are 22-nucleotide noncoding RNAs that are aberrantly expressed in cancer cells and have gained serious consideration as non-invasive cancer biomarkers. We investigated possible differential miRNA signatures that may differentiate high-grade ccRCC from primary disease stages. High-throughput miRNAs expression profiling, using TaqMan OpenArray Human MicroRNA panel, was performed in a group of 21 ccRCC patients. The obtained data was validated in 47 ccRCC patients. We identified nine dysregulated miRNAs (miRNA-210, -642, -18a, -483-5p, -455-3p, -487b, -582-3p, -199b and -200c) in tumor ccRCC tissue compared to normal renal parenchyma. Our results show that the combination of miRNA-210, miRNA-483-5p, miRNA-455 and miRNA-200c is able to distinguish low and high TNM ccRCC stages. Additionally, miRNA-18a, -210, -483-5p and -642 showed statistically significant differences between the low stage tumor ccRCC tissue and normal renal tissue. Contrariwise, the high stages of the tumor process were accompanied by alteration in the expression levels of miRNA-200c, -455-3p and -582-3p. Although the biological roles of these miRNAs in ccRCC are not totally clear, our findings need additional investigations into their involvement in the pathogenesis of ccRCC. Prospective studies with large study cohorts of ccRCC patients are important to further establish the clinical validity of our miRNA markers to predict ccRCC.

## 1. Introduction

Renal cell carcinoma (RCC) is renal-epithelial malignancy that accounts for over 90% of kidney cancer and is a cause for cancer related deaths in the world. According to GLOBOCAN, 431,288 individuals were diagnosed with renal cancer in 2020, which was more than 2% of all cancer diagnoses [1]. Age standardized (world) incidence rates of renal cancer in 2020 were 6.1 per 100,000 in women and 3.2 per 100,000 in men. Most cases of RCC are diagnosed incidentally on magnetic resonance imaging (MRI), computed tomography (CT) or ultrasonography (USG), and only 10% of patients demonstrate classic symptoms of RCC—hematuria, flank pain and palpable masses, so the disease is often detected at the advanced stages [2]. GLOBOCAN statistics report about 179,368 deaths from kidney cancer in 2020, which amount to 1.8% of all cancer deaths worldwide [3].

The vast majority (90%) of RCCs belong to three histological subtypes of clear cell, papillary and chromophobe, with clear cell being the most common and aggressive. These subtypes have important prognostic and therapeutic-prognostic significance [4]. Clear cell RCC (ccRCC), which accounts for 75% of renal cancer diagnoses, is a renal stem cell tumor, usually in the epithelium of the proximal nephron and tubules, and is most likely to metastasize to the lung, liver and bone [5]. ccRCC is characterized by high molecular genetic heterogeneity, metastatic activity and unfavorable prognosis [6]. A distinctive feature of metastasis in ccRCC is its unpredictability. At the time of the diagnosis of the disease, metastases are detected in about 30% of cases; in about half of the patients the disease becomes systemic later, after surgical treatment [7]. Therefore, it is very important to find biomarkers for early diagnosis and for distinct molecular classification and prognosis of ccRCC. miRNA molecules have such a potential for diagnosing, predicting and molecular profiling of ccRCC. MicroRNA (miRNAs) is a small, non-coding, 19–23 nucleotides long, single-stranded RNA that acts as a post-transcriptional control of gene expression and plays an important role in ccRCC [8]. Aberrant expression of miRNAs can occur in tumor cells and affect differentiation, proliferation and apoptosis. The need for and importance of obtaining more accurate knowledge for mechanisms of ccRCC, and the role of miRNA in RCC, is related to a number of clinical and research areas, such as screening, risk stratification, diagnosis, disease severity and prognosis.

In this study, we aimed to detect potential differentially expressed miRNA between ccRCC and normal samples, based on the analyzed microarray datasets, and to find miRNA signatures useful for ccRCC diagnostics and prognosis.

Recently, expression analysis of 723 miRNAs was performed in patients from Fox Chase Cancer Center, which is a National Cancer Institute-designated Comprehensive Cancer Center research facility and hospital located in the Fox Chase section of Philadelphia, Pennsylvania, United States. As a result of this work, specific signature of upregulated miRNA-21-5p, -142-3p, -let-7g-5p, -let-7i-5p, -424-5p and downregulated miRNA-204-5p, associated with ccRCC progression, was shown [9]. Another study, using miRNA GeneChip, demonstrated 31 upregulated miRNAs and 196 downregulated miRNAs in metastatic ccRCC patients from the Chinese People’s Liberation Army General Hospital. The most significantly upregulated miRNAs were miRNA-642b-5p, -3154, -139-5p and downregulated miRNA-891a-5p, -938 and -4271 [10].

Currently, available data on the analysis of microRNA expression in ccRCC tissues are quite numerous, but they are contradictory. In addition, the Volga-Ural region is still the white spot of miRNA expression profiling in ccRCC. In this regard, we decided to perform a high-throughput screening of microRNA expression using microarrays on normal renal parenchyma and tumor ccRCC tissues, grouped by stage, grade or disease progression to identify miRNAs involved in ccRCC development and progression.

## 2. Results

### 2.1. Clinical Characteristics

This study included 68 patients with clear cell renal cell carcinoma. These patients were divided into 2 groups of 21 and 47 patients for miRNA profiling analysis and validation, respectively (Table 1). The gender of profiling group consisted of 10 males (47.6%) and 11 females (52.4%) with the mean age of 59. According to TNM classification, the profiling group consisted of 11 individuals with 1–2 stages of ccRCC and 10 with 3–4 stages. The validation group included 26 males (55.3%) and 21 females (44.7%). The mean age of individuals was 60. The group included 32 cases of 1–2 TNM stage of ccRCC and 15 cases of 3–4 TNM stage. TNM stages 1 and 2 were categorized as low stage (LS) and stages 3 and 4 as high stage (HS) carcinoma. Fuhrman grades 1 and 2 were categorized as low-grade carcinoma (LG). Grades 3 and 4 were grouped as high-grade carcinoma (HG).

### 2.2. miRNA Expression Profiling Analysis

We performed miRNA profiling analysis of ccRCC patients using TaqMan OpenArray Human miRNA Panel (Thermo Fisher Scientific, Waltham, MA, USA,) and showed significant upregulation of miRNA-210, miRNA-642, miRNA-18a, miRNA-483-5p and downregulation of miRNA-455-3p, miRNA-487b, miRNA-29a, miRNA-424, miRNA-199b, miRNA-582-3p, miRNA-375, miRNA-196b, miRNA-200c, miRNA-409-3p in tumor tissue compared to normal renal parenchyma. The relative expression of miRNAs is presented in Table 2.

**Figure 1:**
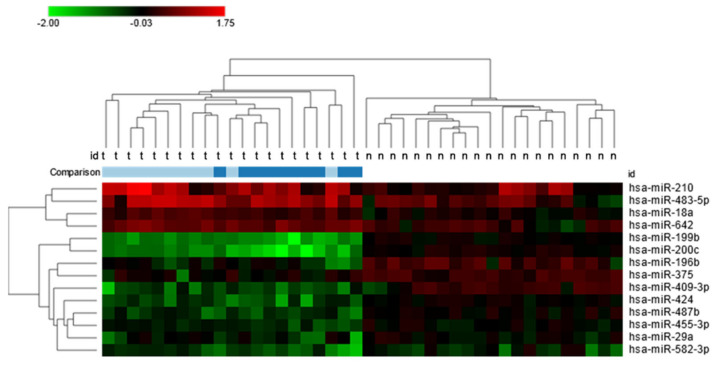
Heat map of 14 significantly, differentially expressed miRNAs between clear cell renal cell carcinoma tissues and normal kidney parenchyma with a fold-change greater than 1. Additionally presented comparison of expression levels between low and high TNM stages: light blue—1–2 TNN stage; dark blue—3–4 TNM stage.

Comparison between low and high TNM stages of the disease showed that upregulated miRNAs characterized tumors of the 1–2 stage, and downregulated miRNAs characterized tumors of the 3–4 stage (Figure 1).

### 2.3. Validation Group miRNA Expression Analysis

The qRT-PCR analysis was applied to verify the 14 candidate miRNAs in the profiling stage with 47 ccRCC patients. Validation analysis showed statistically significant differences of miRNAs expression levels of nine miRNAs: miRNA-210, miRNA-642, miRNA -18a, miRNA -483-5p, miRNA-455-3p, miRNA-487b, miRNA-582-3p, miRNA-199b and miRNA-200c (Table 3; Figure S1).

**Figure 2:**
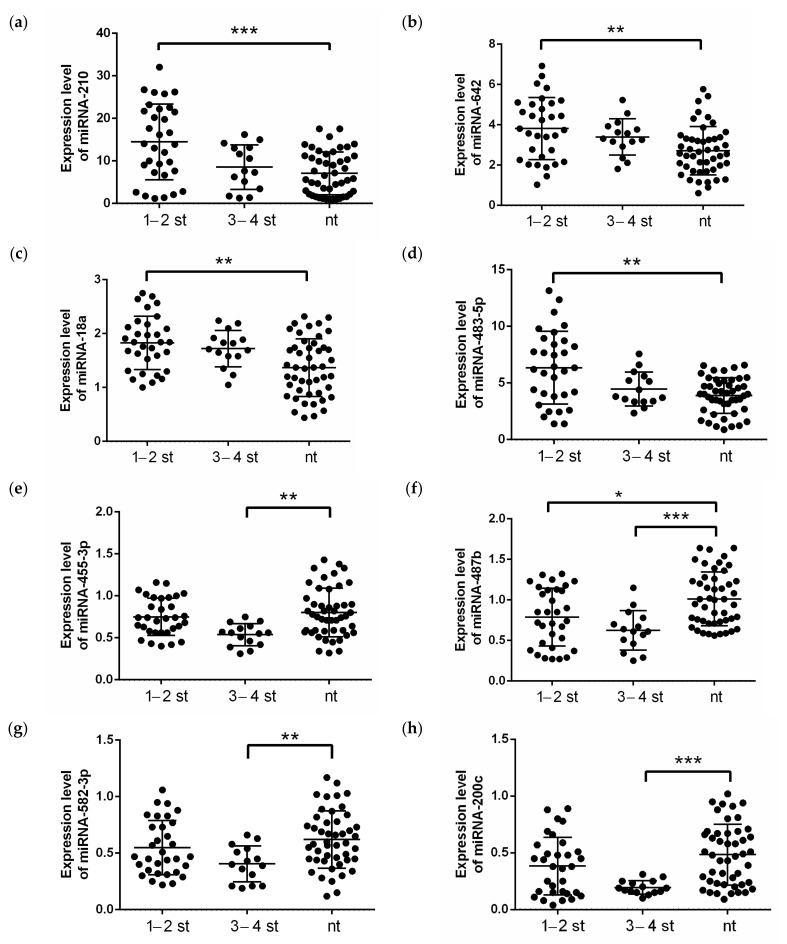
Expression levels of (**a**) miRNA-210, (**b**) miRNA-642, (**c**) miRNA-18a, (**d**) miRNA-483-5p, (**e**) miRNA-455-3p, (**f**) miRNA-487b, (**g**) miRNA-582-3p and (**h**) miRNA-200c in clear cell renal cell carcinoma patients from validation cohort, depending on the stage of the disease. The analysis was performed using qRT-PCR with RNU6, RNU44 and RNU48 as endogenous controls and determined the relative expression of individual miRNAs. *p*-value: ***—*p* < 0.0001, **—*p* < 0.001, *—*p* < 0.01, calculated using ANOVA and nonparametric Kruskal-Wallis test. nt—normal tissue; 1–2 st—TNM stage 1–2, 3–4 st—TNM stage 3–4.

A comparison of miRNAs expression level in renal tissue of low and high tumor stages showed the statistically significant difference in the miRNA-18a, -210, -483-5p and -642 levels (Figure 2a–d) between the low stage tumor ccRCC tissue and normal renal tissue. However, the high stages of the tumor process were accompanied by a change in the expression levels of miRNA-200c, -455-3p and -582-3p (Figure 2e,g,h). The expression levels of miRNA-487b (Figure 2f) were altered in the tumor tissue of the kidney compared to normal tissue in both groups in the low and high stages of the tumor process.

Moreover, we investigated whether there was a significant correlation between the expression levels of selected miRNAs. As shown in Table 4, there is positive and negative correlation between four miRNA pairs.

### 2.4. Logistic Regression and ROC Curve Analyses

We used a stepwise logistic regression method to identify the list of markers distinguishing tumor tissue from normal parenchyma in clear cell renal cell carcinoma. As a result, eight significant miRNAs were included in the logistic regression model (Table 5).

**Figure 3:**
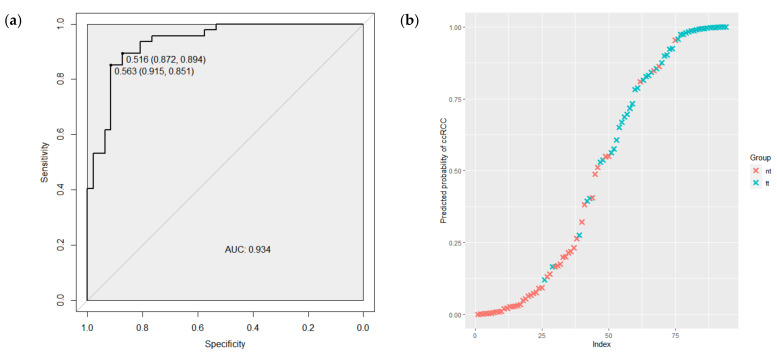
(**a**) The ROC curve regarding the significance expression level of the 8 miRNAs combination to discriminate ccRCC tumor and normal renal tissues. (**b**) The predicted probability of the model to identify tissues as tumors, along with their actual tumor status. ROC—receiver operating characteristic; AUC—area under the curve; tt—tumor tissue; nt—normal renal tissue.

ROC curve analyses were performed to evaluate the capability of miRNAs expression level to discriminate between tumor and normal renal tissues and between stages of tumor process. As shown in Figure 3a, according to the ROC curve analysis, the AUC value of 8 significantly expressed miRNAs combination was 0.934 (95% CI 0.886–0.982), with the sensitivity of 0.894 and the specificity of 0.872. Figure 3b demonstrates the predicted probabilities that tissues are tumors, along with their actual tumor status. The blue crosses presented the samples of ccRCC tumors and, as is shown, most of these have a high probability of being tumors.

In addition, the logistic regression method was used to identify the list of markers distinguishing low and high TNM ccRCC stages. First, we estimated the difference of miRNA expression levels between 1–2 and 3–4 TNM stages and found that significant alteration shows miRNA-210, miRNA-483-5p, miRNA-455 and miRNA-200c. A combination of these miRNAs was estimated using logistic regression analysis (Table 6).

**Figure 4:**
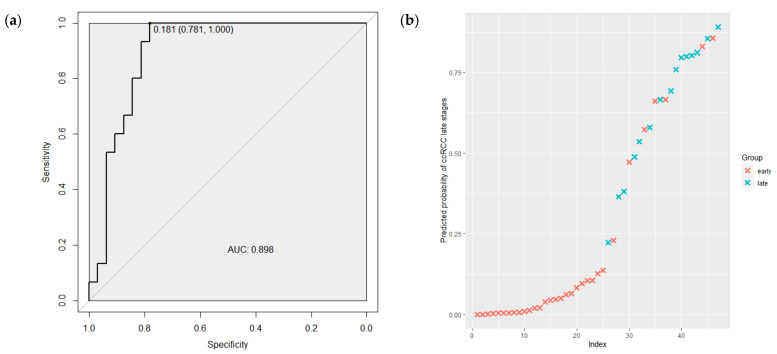
(**a**) The ROC curve regarding the expression level of the miRNAs combination to discriminate ccRCC tumor TNM stages. (**b**) The predicted probabilities of the model to discriminate 1–2 from 3–4 TNM stages. ROC—receiver operating characteristic; AUC—area under the curve; early—1–2 TNM stages of ccRCC; late—3–4 TNM stages of ccRCC.

Furthermore, the ability of this model to discriminate low and high TNM ccRCC stages was tested using ROC curve analysis. As shown in Figure 4a, the AUC for the combination miRNA-210, miRNA-483-5p, miRNA-455 and miRNA-200c was 0.898 (95%CI 0.808–0.988) with a sensitivity of 1.00 and a specificity of 0.78. The predicted probabilities of the model to discriminate 1–2 from 3–4 TNM stages are presented in Figure 4b. As is demonstrated in the graph, the most tumor samples of 1–2 TNM stages (red crosses) have a low probability of belonging to the late class of 3–4 TNM stage tumors.

### 2.5. Expression of Exosomal miRNA-210

**Figure 5:**
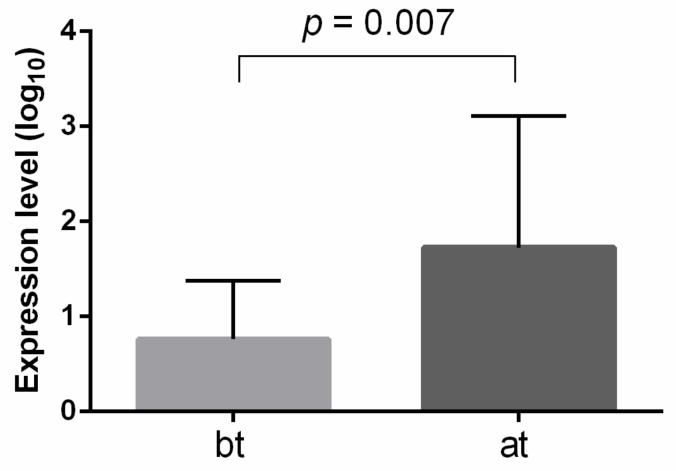
Exosomal miRNA-210 expression level before and after ICI therapy of ccRCC patinets. The analysis was performed using qRT-PCR, with miRNA-16 and hsa-miRNA-1228 as endogenous controls. Significance level *p*-value was calculated using Mann-Whitney U-test. bt—before therapy; at—after therapy.

miRNA-210 is the most well-known miRNA to be engaged in renal cancer development, especially due to its hypoxia and inflammation involvement. We hypothesized that levels of miRNA-210, located within exosomes, could be associated with RCC therapy. Thus, we performed an analysis of exosomal miRNA-210 from the peripheral blood of 35 ccRCC patients, collected before and after immune checkpoint inhibitor therapy (ICI). It was shown that the exosomal miRNA-210 level was significantly higher after ICI therapy compared to its level before therapy initiation (Figure 5).

### 2.6. Expression Analysis of miRNA Target Genes Based on TCGA Data

**Figure 6:**
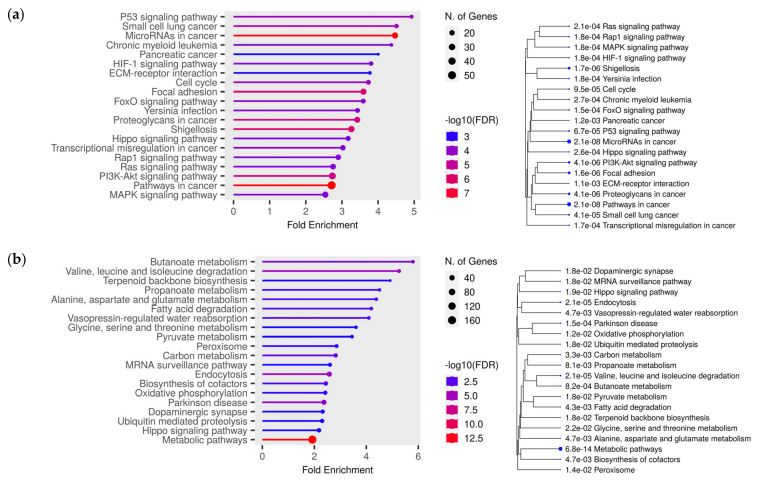
Lollipop charts and hierarchical clustering trees summarizing the correlation among significant pathways for upregulated target genes (**a**) and downregulated (**b**) target genes from TCGA data (KIRC project), from KEGG pathways enrichment analyses, performed using ShinyGO. N. of Genes—number of genes. Color of dots at the lollipop charts indicates the size of enrichment test FDR value (Fisher’s exact test). Bigger dots at the hierarchical clustering tree indicate more significant *p*-values.

Additionally, we performed bioinformatic analysis of RNA-Seq data from TCGA for clear cell kidney cancer (KIRC project) to identify the expression level of target genes of differentially expressed miRNAs from our study. After selecting patients from the European population, the sample size was 52 paired samples (normal/tumor). For each miRNA, a few tens of targets—from 33 to 1912—was observed (Supplementary File S1), with Mann–Whitney *p* < 0.05. Next, target genes with negative correlation to miRNAs expression were subjected to enrichment analysis using the ShinyGO online tool. The results of the enrichment for each set of miRNAs targets are presented in Supplementary Figures S2–S9. In addition, we analyzed two sets of target genes, one of which included all downregulated targets; another set included all upregulated target genes of studied miRNAs. It was observed that, for upregulated target genes, the most significant pathways included P53 signaling pathway, microRNAs in cancers, pancreatic cancer and HIF-1 signaling pathway (Figure 6a). As for downregulated targets, the most enrichment pathways included (Figure 6b) butanoate metabolism, valine, leucine and isoleucine degradation and propanoate metabolism.

## 3. Discussion

miRNAs are a class of small non-coding RNAs that perform posttranscriptional regulation of target genes and have gained serious consideration as cancer biomarkers [11]. In the current study, we profiled miRNAs in ccRCC tumors and investigated the ability of the miRNAs panel to discriminate tumor tissue from normal renal parenchyma. Additionally, we examined whether exosomal miRNAs level could be associated with immune checkpoint inhibitor therapy.

We identified nine miRNAs (miRNA-210, miRNA-642, miRNA-18a, miRNA -483-5p, miRNA-455-3p, miRNA-487b, miRNA-582-3p, miRNA-199b and miRNA-200c) that were dysregulated in tumor ccRCC tissue and normal renal parenchyma. Our results show that the combination of these miRNAs can distinguish between tumor and normal renal tissue, with high sensitivity and specificity. Moreover, a combination of miRNA-210, miRNA-483-5p, miRNA-455 and miRNA-200c is able to distinguish low and high TNM ccRCC stages. This opens up the potential of using miRNAs as additional biomarkers for ccRCC diagnostic and prognosis.

Our results showed upregulation of miRNA-210, miRNA-642, miRNA -18a and miRNA -483-5p in ccRCC tumors compared to normal renal parenchyma. In support of our results, upregulation of miRNA-210 in tumor ccRCC tissue compared to normal kidney parenchyma was observed in patients who are residents of the Czech Republic [12]. There is a lot of evidence of miRNA-210 contribution to cancer development, including ccRCC, suggesting its important role in tumor genesis [13]. It is well known that the main function of miRNA-210 in cancer is connected with its hypoxia involvement. Previously, it was described that miRNA-210 has an anti-apoptotic effect under hypoxic conditions. The main mechanism for increasing the formation of free radicals during hypoxia is associated with the targeting of miRNA-210 to ISCU (iron-sulfur cluster scaffold homolog), which leads to a decrease in the activity of Krebs cycle enzymes and the function of mitochondria. In turn, this stimulates cell survival by inducing a switch to glycolysis during normoxia and hypoxia, and enhances the uptake of iron required for cell growth [14,15]. miRNA-210 hypoxia is supposed to be regulated by acting on hypoxia-induced factor alpha through the direct glycerol-3-phosphate dehydrogenase 1-like (GPD1L) [16]. Numerous studies have demonstrated the presence of microRNA-210 in peripheral blood, which is part of exosomes, microvesicles and apoptotic bodies. It has previously been shown that circulating and exosomal miRNA-210 was upregulated in ccRCC patients compared to controls. However, most interestingly, there was a significant decrease of this miRNA expression in serum samples after surgical tumor resection [17,18]. On the other hand, depletion of miRNA-210 in RCC cell lines resulted in increasing tumorigenesis, which suggested a dual role of miRNA-210 in cancer development and progression [11].

Currently, FDA-approved immuno-oncological drugs are used as monotherapy or in combination with other groups of drugs for the treatment of a wide range of cancers, including ccRCC. Despite the improvement in survival prognosis and a wide variety of treatment regimens with this group of drugs, only a small proportion of patients show a response to therapy; the majority remain refractory and resistant to treatment [19]. In this connection, it is necessary to search and develop specific markers of the effectiveness of therapy, where microRNAs are also highly promising. In the current study, we observed increasing exosomal miRNA-210 after immune checkpoint inhibitor therapy in ccRCC patients. Recently, it was shown that miRNA-210 expression could be regulated by a mechanism independent of HIF1-alpha in infiltrated immune cells of the triple-negative breast cancer [20]. To date, there is much evidence suggesting that miRNA-210 might modulate the immune response. It was shown that miRNA-210 could induce IL-17/IL-17A production, as well as attenuate the immune regulatory functions of Tregs [21]. It was also observed that overexpression of miRNA-210 inhibits FOXP3 expression and impairs the immunosuppressive functions of Treg cells in CD4+ T cells. Moreover, CD4+ T cells, transfected with miRNA-210 inhibitor, demonstrated upregulation of IL-10 and TGF-β mRNA and downregulation of IFN-γ and IL-17A mRNA with negative control [22]. While it is known that IL-10 is one of the cytokines activating STAT3—a well-known activator of transcription that plays an important role of renal cell carcinoma development—increased STAT3 activation correlates with both advanced metastatic disease and worse survival in RCC [23]. It was shown that downregulation of miRNA-210-5p decreased phosphorylated STAT3, whereas miRNA-210-5p overexpression did the opposite, indicating that miRNA-210-5p is involved in STAT3-related signaling in response to TGFβ stimulation [19,24]. Another member of JAK/STAT pathway—STAT5—has been shown to play a vital role in immune cells, and an absence of lymphoid STAT5A leads to a loss of CD8+ and regulatory T cells (Treg). In addition, STAT5 has been identified as a cytosolic signaling molecule involved in proliferation, differentiation and apoptosis in various cell types. Interestingly, STAT5A was found as a direct target of miRNA-210-5p. In vivo experiments showed that STAT5A knockdown, combined with radiotherapy, was associated with greater numbers of CD8+ T cells and fewer Tregs [25].

Recent studies have reported the low abundance of miRNA-642 in various human tissues and cell lines [26]. miRNA-642 downregulation reduced the cell viability in bladder cancer cell lines, with different cisplatin sensitivity [27]. It was described recently that miRNA-642 could act as a tumor suppressor through regulating p38 MAPK signaling pathway in hepatocellular carcinoma [28]. In addition, miRNA-642 served as a tumor suppressor in prostate cancer. It was shown that overexpression of miRNA-642 in prostate cancer cells resulted in reduced cell viability by targeting deoxyhypusine hydroxylase (DOHH) that regulates cell growth through the eukaryotic translation initiation factor. The tumor suppressor features of miRNA-642 was also confirmed by an alteration of expression of potential target genes, such as WT1, NUAK1, RASSF3, SKP2, GPS2 and IGFBP3, in prostate cancer [29]. Furthermore, miRNA-642 was described as downregulated in colorectal cancer cells, in peripheral immune cells following resection of lung tumors, in childhood hematological cancers and hepatocellular carcinoma [28,30,31].

Several studies indicated that miRNA-18a is mostly upregulated in different types of cancer, including renal cell carcinoma [32,33]. miRNA-18a belongs to the miRNA-17-92a cluster and is transcribed from a polycistronic non-protein-coding gene. It is well known that miRNA-18a is involved in cancer progression and its level correlates with tumor size and TNM stage [34]. miRNA-18a could activate the mTOR pathway by targeting SMG1 and influence cell survival, epithelial-to-mesenchymal transition (EMT) and invasion. It is supposed that miRNA-18a may only be involved in the initial steps of tumor progression, such as invasion, and its role in further progression is limited [35]. Additionally, *HIF1α* and *PVT1* were believed to be potential targets of miRNA-18a. Interestingly, the expression of *HIF1A* had a significantly negative correlation with *PVT1* in ccRCC samples [34]. It was shown that RCC patients with low expression of miRNA-18a had significantly better overall survival [27,32], while upregulation of miRNA-18a-5p had a positive effect on RCC cell proliferation, migration, invasion and inhibition of apoptosis [33]. In addition, in sunitinib-resistance RCC cell lines, the level of miRNA-18a was significantly reduced [36].

We observed upregulation of miRNA-483-5p in RCC tissue compared with normal renal parenchyma. Interestingly, Wang X. et al. reported downregulation of miRNA-483-5p in renal tumor tissues, but the level of this miRNA was upregulated in plasma of RCC patients after nephrectomy [37]. However, in Wilm’s tumor mouse model, an anti-tumor effect of miRNA-483-5p, a mimic of which markedly induced tumor cell apoptosis via downregulation of MKNK1 and upregulation of cleaved caspase 3 and cleaved PARP levels, was shown. In addition, low expression of miRNA-483-5p was significantly associated with unfavorable histology subtype, lymphatic metastasis and late clinical stage [38].

A large cohort analysis based on the TCGA database showed that low expression levels of miRNA-455-5p and miRNA-455-3p were associated with poor survivals in RCC patients. Yamada Y. et al. identified 33 targets of miRNA-455-3p, several of which have been reported to be involved in RCC development, contributing to cancer cell proliferation, invasion and migration. Thus, it was believed that miRNA-455-3p acted as anti-tumor miRNAs through their targeting of several oncogenes in RCC [39].

To the best of our knowledge, miRNA-487b has not been described in renal cancer. However, miRNA-487b was shown to be downregulated in osteosarcoma CD133 positive cell lines [40] and inhibited osteosarcoma chemoresistance and metastasis [41]. The suppressive role of miRNA-487b was also indicated in colorectal cancer via targeting MYC, SUZ12 and KRAS [42]. On the other hand, in hepatocellular carcinoma, higher miRNA-487b expression was observed in the serum of HCC patients compared with healthy individuals, and was associated with poor prognosis [43].

Knowledge about the role of miRNA-582-3p in renal cell carcinoma is limited. However, it was shown that miRNA-582-3p was downregulated in prostate cancer [44], acute myeloid leukemia [45] and hepatocellular carcinoma [46], and overexpressed in cervical cancer [47] and hypoxia-related lung cancer [48]. miRNA-582-3p could regulate the WNT/β-catenin signaling pathway, thereby blocking the progression of cancer.

miRNA-199b was shown to be downregulated in RCC tissues and cell lines. In addition, the inhibition of miRNA-199b promoted proliferation and reduced the apoptotic rate of tumor cells, suggesting a tumor suppressor role of this miRNA [49]. One of the potential targets of miRNA-199b is *DDR1* (discoidin domain receptor tyrosine kinase 1), which demonstrated oncogenic role in ccRCC [50]. Furthermore, miRNA-199b might serve critical roles in the regulation of immune response via targeting *PPARGCA1*—the encoding gene of PPARG coactivator 1α, which is a co-regulator of mitochondrial biogenesis and oxidative phosphorylation [51].

miRNA-200c is a member of the miRNA-200 family and the most frequently downregulated miRNA in ccRCC [52,53]. It was shown that miRNA-200c plays an important role in epithelial to mesenchymal transition by inducing E-cadherin expression [54]. Moreover, R. Saleeb et al. showed that miRNA-200b, and miRNA-200c-positive ccRCC patients, have a statistically significant lower chance of disease-recurrence or relapse, and multivariate analyses showed that miRNA-200b and miRNA-200c-positive patients had longer disease-free survival [52]. Overall, survival analysis using the Cancer Genome Atlas data revealed that miRNA-200b-positive patients have significantly better survival. Their results also suggest that miRNA-141, miRNA-200b and miRNA-200c may be used as independent prognostic markers for ccRCC.

## 4. Materials and Methods

### 4.1. Tumor Samples and Data Collection

Paired samples of tumor and normal renal tissues were obtained from clear cell renal cell carcinoma patients treated in the Republican Clinical Oncological Dispensary, Departments of Oncology and Urology of the Clinic of Bashkir State Medical University, between 2014 and 2020. These tissues were examined by 2 independent pathologists experienced in RCC for histological cell type, TNM stages and Fuhrman nuclear grade. Informed consent was obtained from each patient for the collection of biological material and molecular genetic studies. The study protocol was approved by the Research Ethics Committee of the Institute of Biochemistry and Genetics—Subdivision of the Ufa Federal Research Center of the Russian Academy of Sciences and conducted according to the ethical standards of the Bioethics Committee developed by the Declaration of Helsinki World Association. High-throughput miRNAs expression profiling using TaqMan OpenArray Human MicroRNA panel was performed in a group of 21 ccRCC patients. For the validation stage, a set of 47 ccRCC patients were used. The criterion for inclusion of all patients in the study was a histologically confirmed diagnosis of clear cell renal cell carcinoma and 80% minimum of tumor cells in each tumor sample. Clinical data included gender, age, TNM stage, histological type and Fuhrman grade. Moreover, such patients were not prescribed chemotherapy or radiotherapy prior to the collections of tissue samples. There were no age, sex, ethnicity or cancer stage restrictions for participation in the study.

### 4.2. miRNA Extraction, Reverse Transcription and Expression Analysis of 754 miRNAs

**Figure 7:**
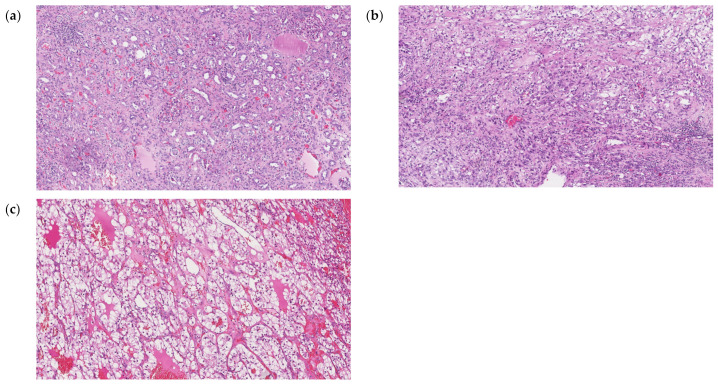
Hematoxylin and eosin (H&E) staining histopathology of normal kidney parenchyma (**a**), ccRCC G2 (**b**) and ccRCC G3 (**c**) tissues. Each image are the views under 100× magnification.

Frozen tumor tissue sections were stained with H&E and examined by 2 pathologists in order to choose the region with ≥80% tumor cell content (Figure 7) to be used for RNA extraction. Normal tissues did not contain tumor cells. Total RNA from tissue samples were isolated using Direct-zol™ RNA MiniPrep (Zymo Research, Irvine, CA, USA), according to protocol. The total RNA was used for further analysis or stored at −70 °C. The concentration and purity of the isolated RNA were determined by measuring optical density using a NanoDrop ND-1000 spectrophotometer (Thermo Scientific, Waltham, MA, USA). The expression levels of 754 miRNAs were profiled using the TaqMan OpenArray Human MicroRNA panels (PN: 4470189; Life Technologies Forster City, CA, USA) on a QuantStudio 12K Flex instrument. For all experimental groups, 3 µL (~10 ng) of total RNA was used for reverse transcription (RT) reactions using MegaPlex RT Primers Human Pool Set v3.0 (PN: 4444745; Pool A v2.1 and Pool B v3.0), according to the manufacturer’s protocol. 

Reverse transcription was performed with the Human TaqMan MicroRNA Reverse Transcription Kit (Life Technologies) using a set of two predefined Megaplex™ RT primer pools (pool A and pool B). According to the manufacturer’s protocol, total RNA for the reverse transcription reaction was taken in an amount of 100 ng. Thus, the reaction mixture for each sample contained:

- 100 ng of total RNA in a volume of 3 μL.
- 4.5 μL reverse transcription mix containing reverse transcriptase, Megaplex™RT Primers Pool A or Pool B and other reverse transcription reagents.

The reverse transcription reaction conditions were as follows: 16 °C—2 min, 42 °C—1 min, 50 °C—1 min during 40 cycles, held at 85 °C—5 min and then kept at 4 °C.

The cDNA products were then subjected to a preamplification reaction using a set of two pools (Pool A and Pool B) of Megaplex™ PreAmp gene-specific forward and reverse primers. The reaction conditions of the preamplification were as follows: 95 °C—10 min, 55 °C—2 min, 72 °C—2 min, and after 12 cycles of 95 °C—15 s and 60 °C—4 min; finally, 99.9 °C—10 min and then kept at 4 °C.

The preamplification product was diluted with RNases/DNases free water in a ratio of 1:40. The reaction mixture for PCR on TaqMan^®^ OpenArray^®^ MicroRNA panels contained 22.5 μL TaqMan^®^ OpenArray^®^ Real-Time PCR Master Mix and 22.5 μL preamplification products for each of the pools, separately. The transfer of samples from a 384-well plate to the OpenArray chip was performed using the AccuFill™ System robotic setup.

### 4.3. miRNA Individual Assays

miRNAs found differentially expressed at the profiling stage were analyzed using individual RT-qPCR assays. Reverse transcription was performed using the TaqMan^®®^ MicroRNA Reverse Transcription Kit (Applied Biosystems, Waltham, MA, USA). The reverse transcription reaction mixture contained 5 μL total RNA, 7 μL master mixture containing reverse transcriptase, dNTPs and other reaction components and 3 μL 5X OT primer specific for the selected miRNA gene. The reverse transcription reaction was carried out under the following conditions: 16 °C—30 min, 42 °C—30 min, 85 °C—5 min, 4 °C—∞. Real time quantitative PCR was performed using the TaqMan MicroRNA Assays kit (Applied Biosystems) and the CFX96™ Real Time PCR Product Detection System (BioRad, Hercules, CA, USA).

### 4.4. Bioinformatic Analysis of the TCGA Data

With the view to identify differentially expressed target genes of studied miRNAs, we performed bioinformatics analysis of the data from TCGA database for clear cell kidney cancer (KIRC project). The European population (Caucasian) was chosen as the most appropriate to compare with the population from our study. For these data, we analyzed differential gene expression between normal and tumor groups using the edgeR package in R [11]. Using the multiMiR package [12], we compiled lists of target genes for selected miRNAs. The lists were formed on the basis of miRecords, miRTarBase and TarBase databases, with experimentally confirmed miRNA-target association. We crossed the lists of target genes for each microRNA with the results of gene expression, leaving only those genes, the expression of which was statistically significantly, which changed in the tumor group (FDR tests > 0.05). Pathway analysis of predicted target genes was performed using the ShinyGO [55] online resource.

### 4.5. Statistical Analysis

Expression Suite Software v.1.0.1 was used for profiling data analysis. Global normalization was used to normalize the data. The global normalization method consists of three consecutive steps. First, all Cq values above a certain threshold are treated as noise and removed from further analysis (we used cycle 26 as the threshold value). Then, the arithmetic mean Cq value for each individual sample is calculated and the arithmetic mean Cq value is subtracted from the Cq value of the individual sample; the Cq values are then converted to relative values (RQs). This procedure results in normalized expression values on the logE scale. To quantify gene expression, the 2^−ΔΔCt^ method was used based on the fact that the difference in the value of the “threshold cycle” (ΔCt) between the gene under study and the control gene is proportional to the level of relative expression of the gene under study. The means of the 3 housekeeping miRNAs (RNU6, RNU44 and RNU48) were used to normalize the expression of each target miRNA. With comparing groups on a quantitative basis, the equality of the variances of the distributions of signs was checked using the Mann-Whitney U-test. ANOVA and Kruskal–Wallis tests were used for the comparisons of genes expression levels for normally distributed and nonparametric data, respectively. Whether the data of any two groups was normally distributed was confirmed using Shapiro–Wilk tests. Statistical significance was measured by *p*-values, controlled for the false discovery rate (FDR), using the Benjamini–Hochberg method to account for multiple testing. Calculations were made using GraphPad Prism 6.07 software (San Diego, CA, USA) and R environment. Discriminatory capacities of logistic regression models were evaluated using receiver operating characteristic (ROC) curves in the R environment with the pROC package. The strength of the model to distinguish tumor tissue from normal parenchyma ccRCC was assessed by comparing the area under the curve (AUC) of the respective ROC curves, which compares the true-positive rate against the false-positive rate.

## 5. Conclusions

In summary, we have identified a miRNA signature that is capable of distinguishing high-grade ccRCC from low-grade disease and has the potential to differentiate initial disease stages from metastatic disease.

Although the biological roles of these miRNAs in ccRCC are not totally unclear, our findings need additional investigations into their involvement in the pathogenesis of ccRCC. Prospective studies with large study cohorts of ccRCC patients are important to further establish the clinical validity of our miRNA markers to predict ccRCC.

## Figures and Tables

**Table 1:** Demographic data and clinical features of the subjects.

	Profiling Group (*n* = 21)	*p*-Value	Validation Group *n* = 47	*p*-Value
Age, mean (range)	59 (43–74)		60 (38–78)	
Gender, *n* (%)				
male	10 (47.6)	0.22	26 (55.3)	0.30
female	11 (52.4)		21 (44.7)	
TNM stage, *n* (%)				
1–2	11 (52.4)	1.00	32 (68.1)	0.001
3–4	10 (47.6)		15 (31.9)	
Fuhrman grade				
1–2	14	0.063	27 (57.4)	0.216
3–4	7		20 (42.6)	

**Table 2:** Differentially expressed microRNA genes in tumor and normal renal tissue of patients with clear cell renal cell carcinoma.

miRNA	Fold Change	P_FDR_-Value
miRNA-18a	2.79	0.023
miRNA-210	3.61	0.034
miRNA-483-5p	2.49	0.044
miRNA-642	4.15	0.021
miRNA-29a	0.21	0.012
miRNA-196b	0.09	0.023
miRNA-199b	0.04	0.021
miRNA-200c	0.03	0.001
miRNA-375	0.04	0.020
miRNA-409-3p	0.15	0.012
miRNA-424	0.19	0.007
miRNA-455-3p	0.36	0.037
miRNA-487b	0.27	0.026
miRNA-582-3p	0.04	0.001

**Table 3:** Differentially expressed miRNAs from the validation group of ccRCC patients.

miRNA	Tumor (Mean ± SEM)	Normal Tissue (Mean ± SEM)	*p*-Value
miRNA-18a	1.79 ± 0.06	1.37 ± 0.08	0.0003
miRNA-199b	0.64 ± 0.04	0.73 ± 0.04	0.040
miRNA-200c	0.32 ± 0.03	0.48 ± 0.04	0.002
miRNA-210	12.58 ± 1.21	7.09 ± 0.73	0.001
miRNA-455	0.68 ± 0.03	0.80 ± 0.04	0.036
miRNA-483-5p	5.75 ± 0.42	3.89 ± 0.23	0.003
miRNA-487b	0.74 ± 0.05	1.01 ± 0.05	0.0003
miRNA-582-3p	0.50 ± 0.03	0.62 ± 0.04	0.02
miRNA-642	3.68 ± 0.20	2.72 ± 0.16	0.001

**Table 4:** Correlation matrix between the microRNAs *.

	miRNA-210	miRNA-642	miRNA-483-5p	miRNA-18a	miRNA-455-3p	miRNA-487b	miRNA-199b	miRNA-582-3p	miRNA-200c
miRNA-210		0.057	0.067	0.088	0.072	−0.102	−0.150	0.092	−0.104
miRNA-642	0.057		0.121	0.115	−0.104	−0.114	−0.203	−0.062	−0.272
miRNA-483-5p	0.067	0.121		0.278	−0.133	−0.247	0.080	−0.306	−0.013
miRNA-18a	0.088	0.115	0.278 ^b^		−0.083	−0.186	0.006	−0.106	−0.234
miRNA-455-3p	0.072	−0.104	−0.133	−0.083		0.072	−0.042	0.278	0.064
miRNA-487b	−0.102	−0.114	−0.247	−0.186	0.072		0.072	0.237	0.219
miRNA-199b	−0.150	−0.203 ^a^	0.080	0.006	−0.042	0.072		0.024	0.068
miRNA-582-3p	0.092	−0.062	−0.306 ^b^	−0.106	0.278 ^b^	0.237 ^a^	0.024		0.114
miRNA-200c	−0.104	−0.272 ^b^	−0.013	−0.234 ^a^	0.064	0.219 ^a^	0.068	0.114	

* Correlation coefficients of all samples, according to Spearman, were calculated using the expression ratios of target microRNAs related to the means of normalizer genes RNU6, RNU44 and RNU48. Significance level: ^a^—*p* < 0.05; ^b^—*p* < 0.01.

**Table 5:** Results of logistic regression analysis for eight significant miRNAs.

Variables	Estimate	Std, Error	z Value	Pr (>|z|)
(Intercept)	−1.3823	2.4403	−0.566	0.571089
miRNA-210	0.1964	0.0593	3.312	0.000925
miRNA-642	0.7983	0.3231	2.471	0.013488
miRNA-483-5p	0.4462	0.193	2.312	0.020785
miRNA-18a	1.7446	0.7545	2.312	0.020754
miRNA-455-3p	−3.7004	1.6417	−2.254	0.024199
miRNA-487b	−2.7879	1.1082	−2.516	0.011883
miRNA-199b	−2.3323	1.3975	−1.669	0.095146
miRNA-200c	−2.5133	1.3097	−1.919	0.054997

**Table 6:** Results of logistic regression analysis for differentially expressed miRNAs in 1–2 vs. 3–4 TNM stages.

Variables	Estimate	Std. Error	z Value	Pr (>|z|)
(Intercept)	8.45921	2.86408	2.954	0.00314
miRNA-210	−0.17216	0.08355	−2.061	0.03934
miRNA-455-3p	−8.05981	3.27772	−2.459	0.01393
miRNA-483-5p	−0.38919	0.28831	−1.350	0.17705
miRNA-200c	−1.22501	3.48936	−0.351	0.72554

## Data Availability

The data used and obtained during the study are available from the corresponding author on request.

## Supplementary Materials

The supporting information can be downloaded at: https://www.mdpi.com/article/10.3390/ijms24086909/s1.
