# Supplementary material for: MicroRNA Expression Signatures in Clear Cell Renal Cell Carcinoma: High-Throughput Searching for Key miRNA Markers in Patients from the Volga-Ural Region of Eurasian Continent
Source: Int J Mol Sci. 2023 Apr 7;24(8):6909. doi: 10.3390/ijms24086909 (PMC10139074; doi:10.3390/ijms24086909)
Supplement: Supplementary file 1 [file ijms-24-06909-s001.zip › Supplementary figures.pdf]

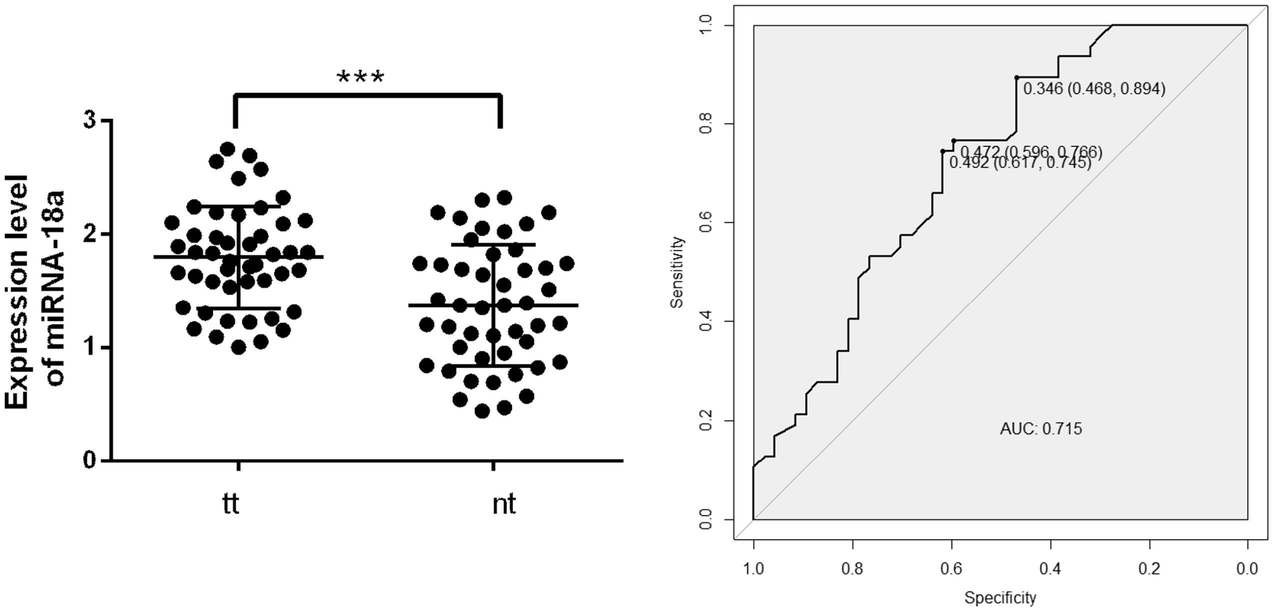
***
3
2
1
0
Expression level of miRNA-18a
tt
nt
1.0
0.8
0.6
0.4
0.2
0.0
Sensitivity
Specificity
0.346 (0.468, 0.894)
0.472 (0.596, 0.766)
0.492 (0.617, 0.745)
AUC: 0.715

a

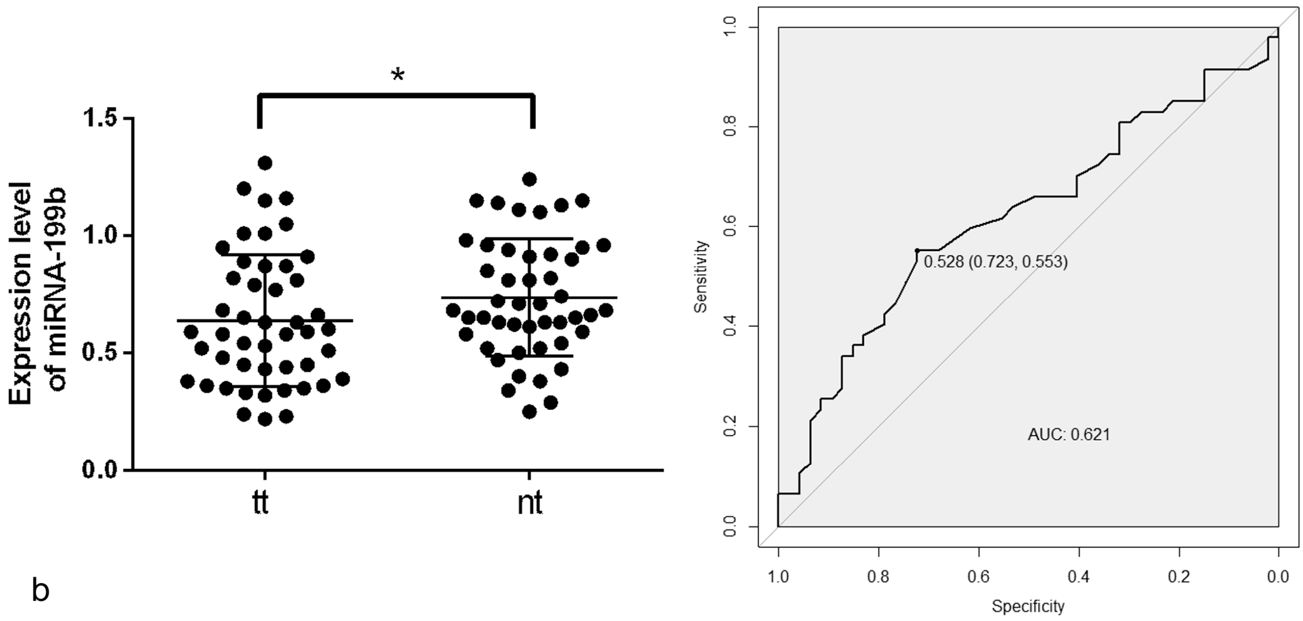
*
1.5
1.0
0.5
0.0
Expression level of miRNA-199b
tt
nt
Sensitivity
Specificity
0.528 (0.723, 0.553)
AUC: 0.621

b

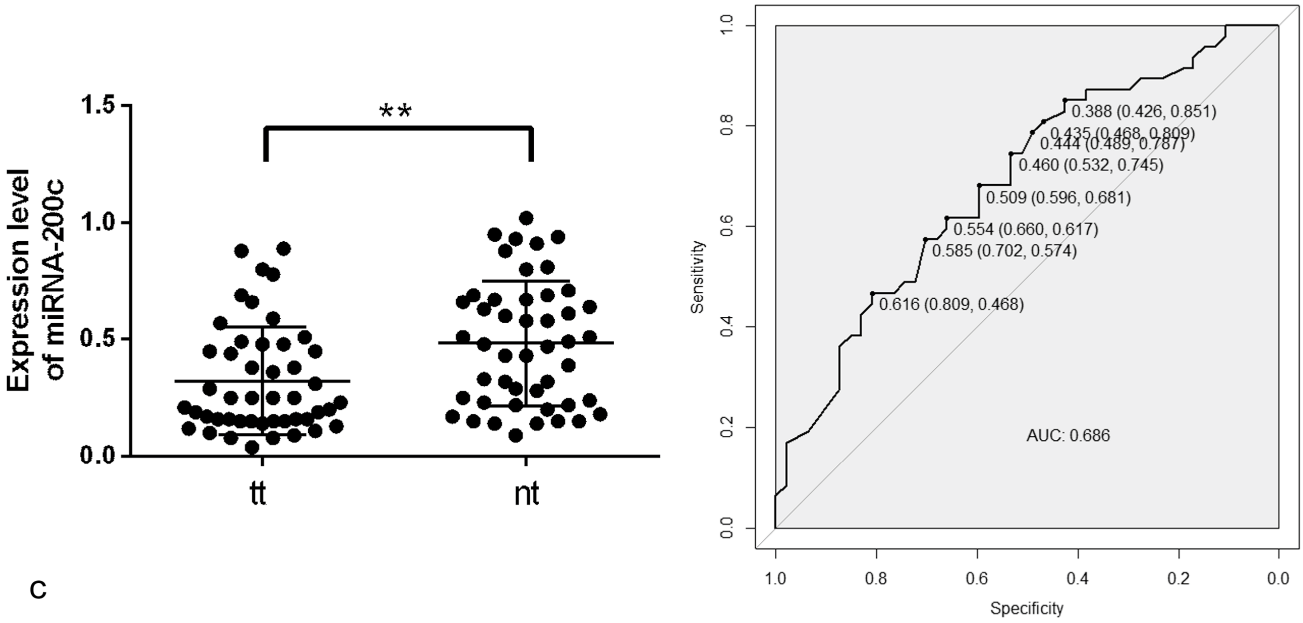
**
1.5
1.0
0.5
0.0
Expression level of miRNA-200c
tt
nt
Sensitivity
Specificity
0.388 (0.426, 0.851)
0.435 (0.468, 0.809)
0.444 (0.489, 0.787)
0.460 (0.532, 0.745)
0.509 (0.596, 0.681)
0.554 (0.660, 0.617)
0.585 (0.702, 0.574)
0.616 (0.809, 0.468)
AUC: 0.686

c

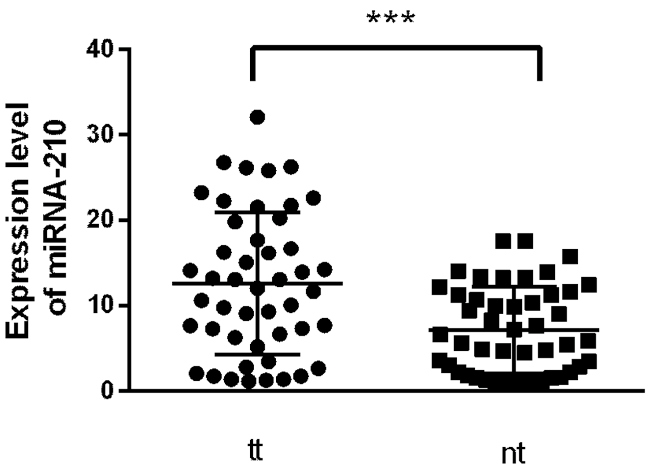
***
40
30
20
10
0
Expression level
of miRNA-210
tt
nt

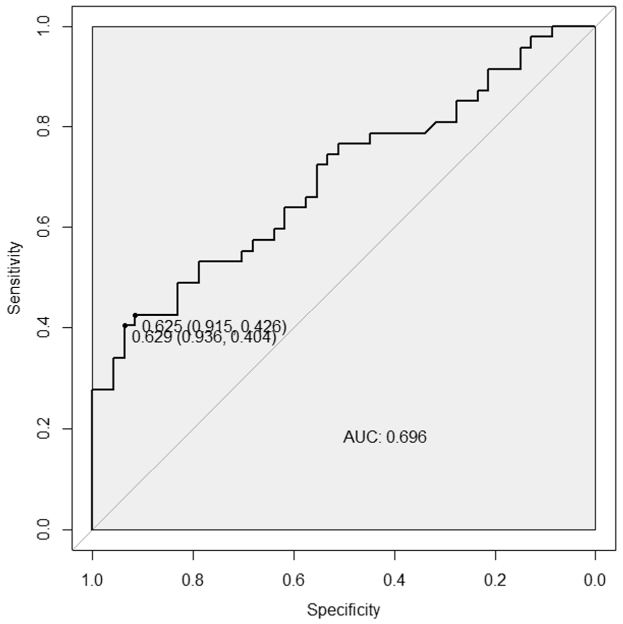
1.0
0.8
0.6
0.4
0.2
0.0
Sensitivity
0.625 (0.915, 0.426)
0.629 (0.936, 0.404)
AUC: 0.696
1.0
0.8
0.6
0.4
0.2
0.0
Specificity

d

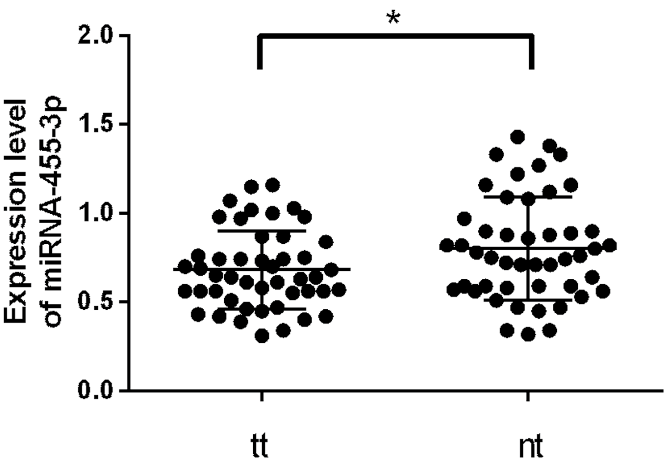
*
2.0
1.5
1.0
0.5
0.0
Expression level
of miRNA-455-3p
tt
nt

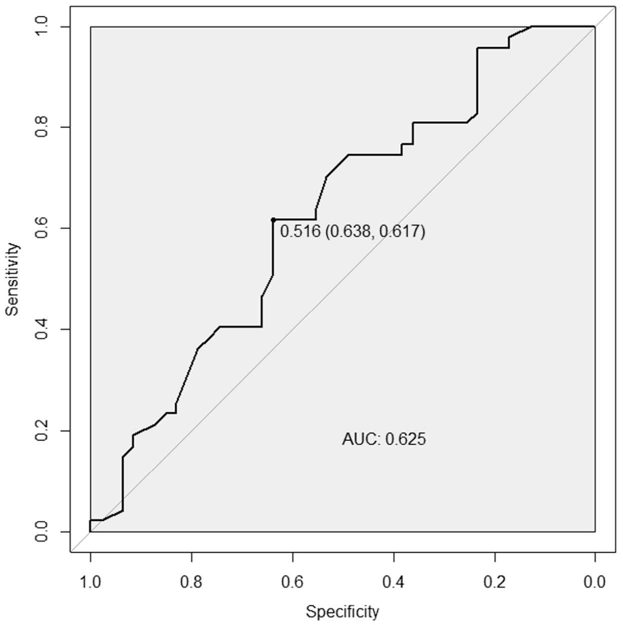
1.0
0.8
0.6
0.4
0.2
0.0
Sensitivity
0.516 (0.638, 0.617)
AUC: 0.625
1.0
0.8
0.6
0.4
0.2
0.0
Specificity

e

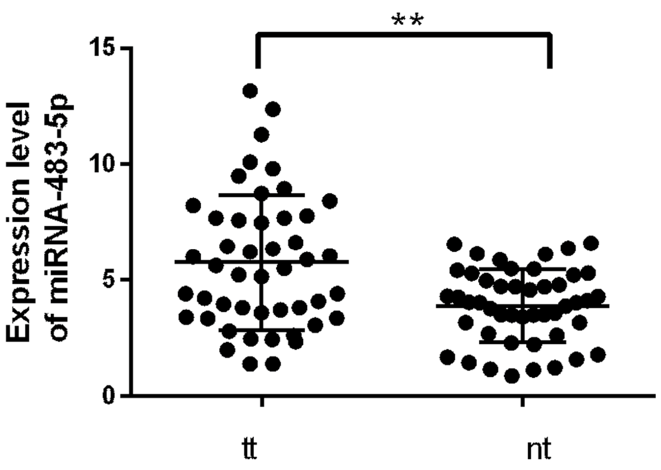
**
15
10
5
0
Expression level
of miRNA-483-5p
tt
nt

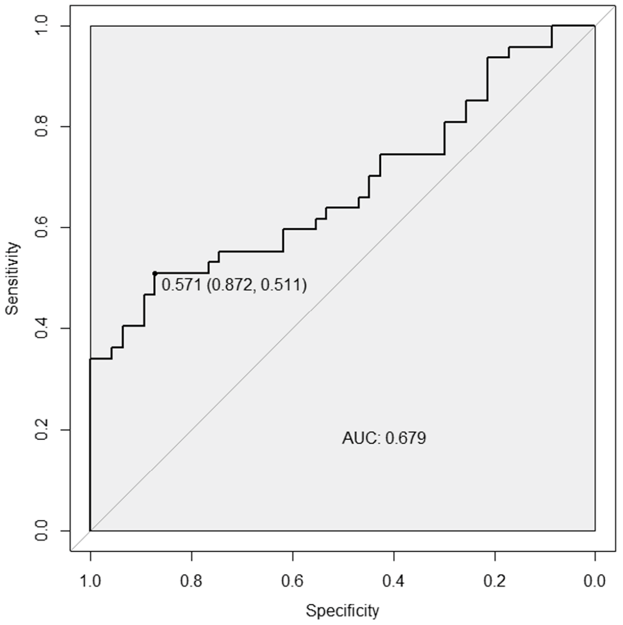
1.0
0.8
0.6
0.4
0.2
0.0
Sensitivity
0.571 (0.872, 0.511)
AUC: 0.679
1.0
0.8
0.6
0.4
0.2
0.0
Specificity

f

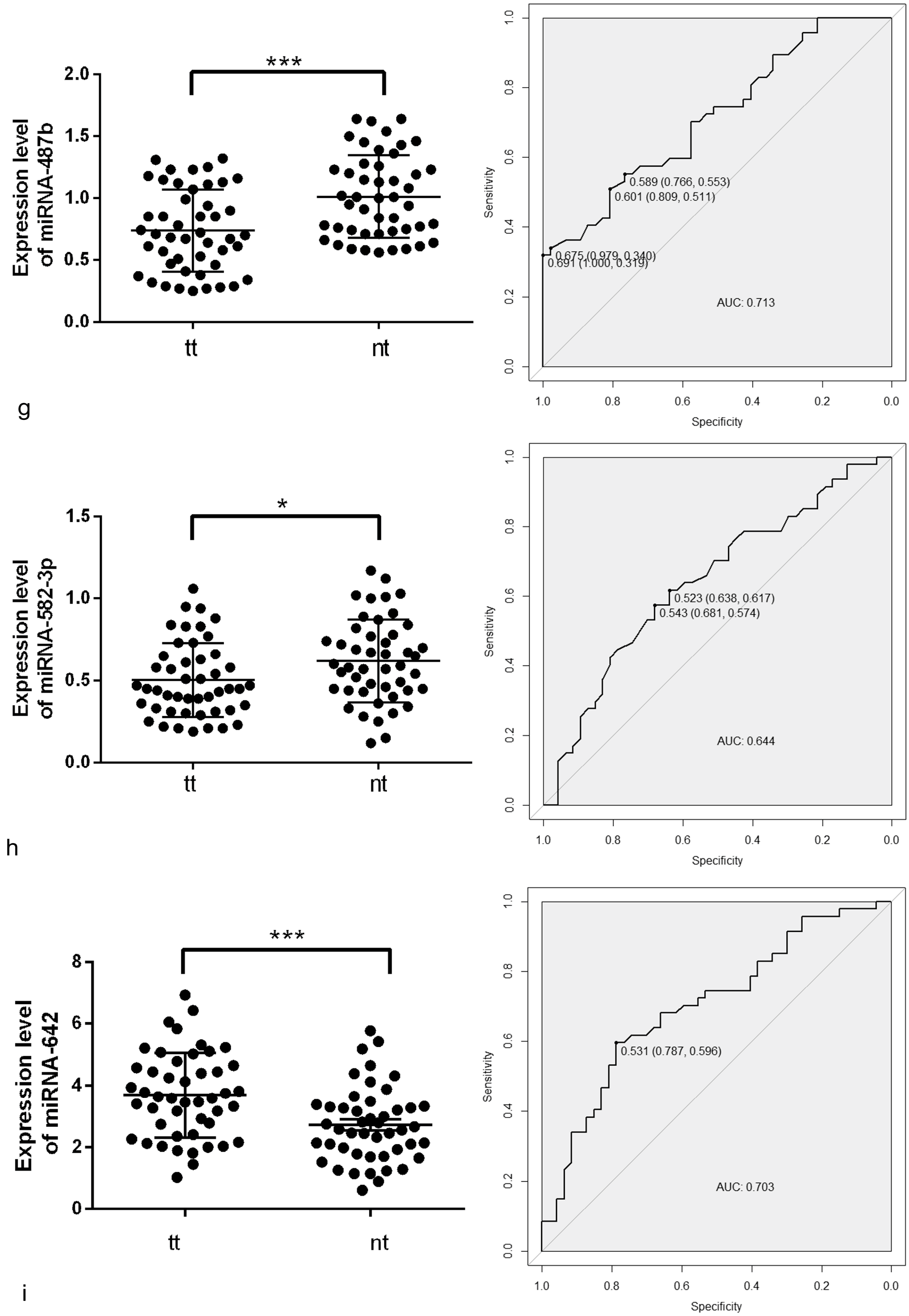

Figure S1: MicroRNAs from validation analysis with significant differences. The scatter plots show (**a**) miRNA-18a, (**b**) miRNA-199b, (**c**) miRNA-200c, (**d**) miRNA-210, (**e**) miRNA-455-3p, (**f**) miRNA-483-5p, (**g**) miRNA-487b, (**h**) miRNA-582-3p and (**i**) miRNA-642 expression level analysis performed using Mann–Whitney U test. The line plots present ROC-curve analysis of each miRNAs discrimination power to distinguish tumor and normal tissues. tt—ccRCC tumor tissue; nt—normal renal tissue; AUC—area under the curve; *p*-value: ***—$p < 0.0001$, **—$p < 0.001$, *—$p < 0.01$.

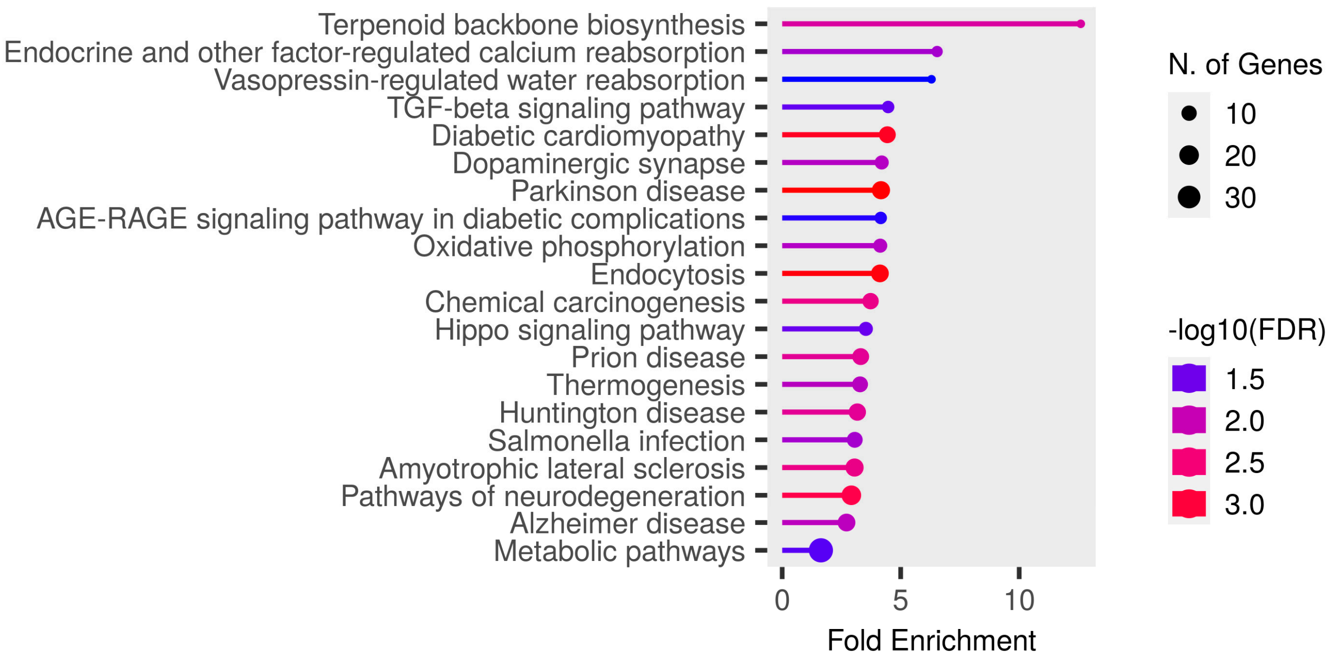

Figure S2: The results of enrichment of miRNA-18a targets.

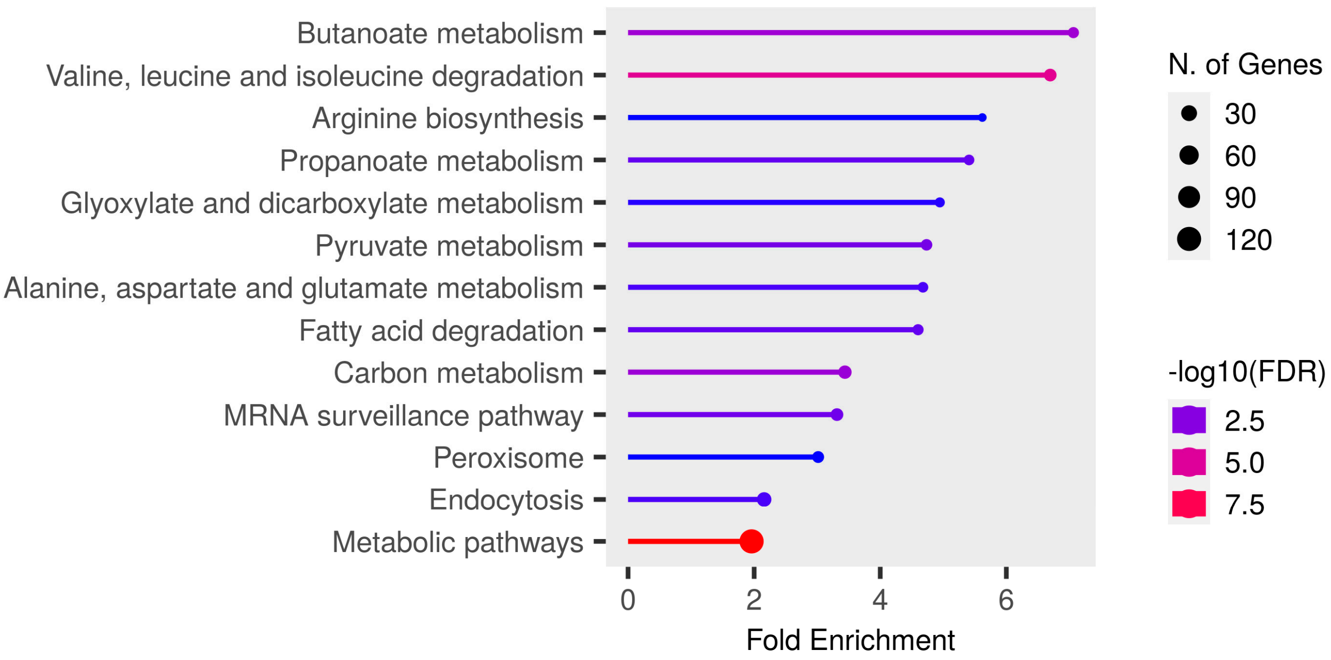

Figure S3: The results of enrichment of miRNA-210 targets.

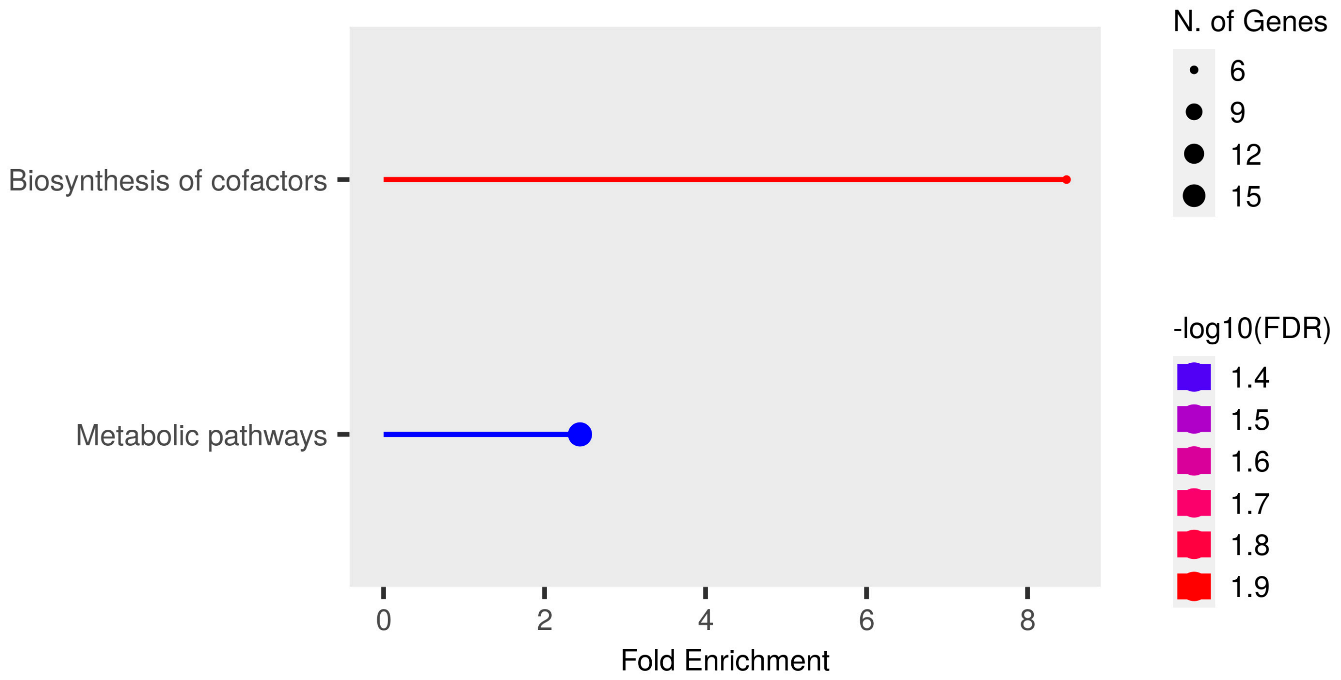

Figure S4: The results of enrichment of miRNA-642 targets.

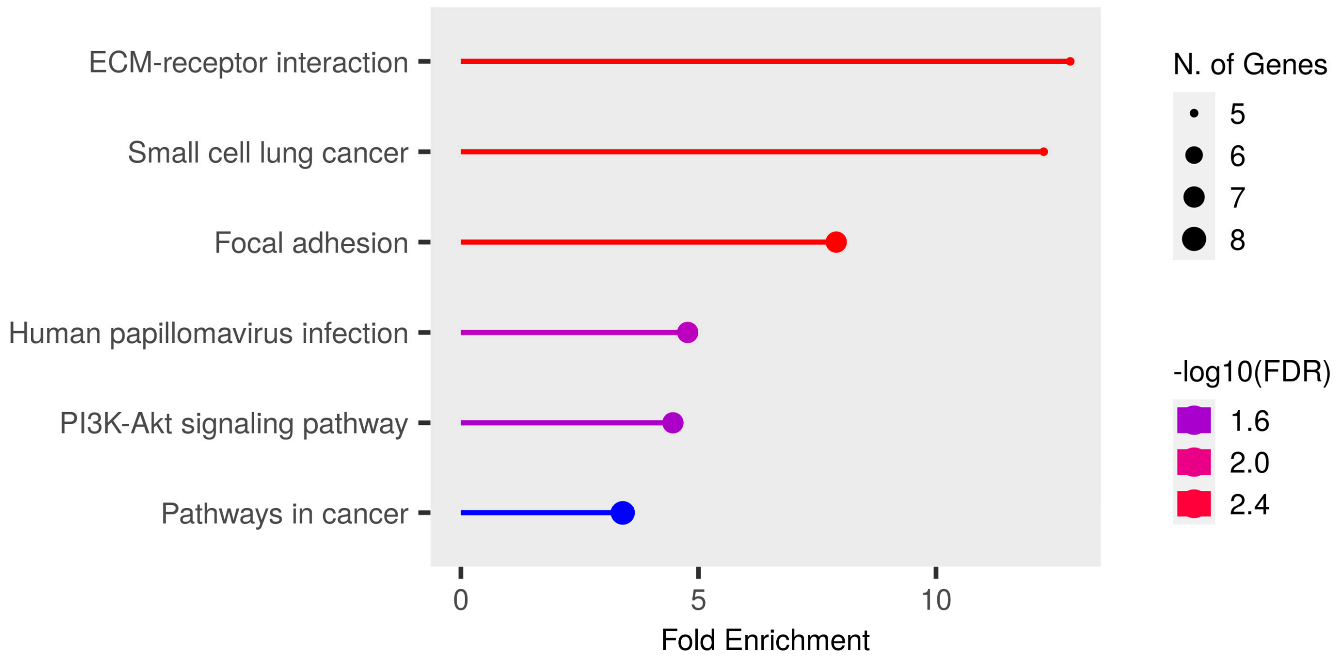

Figure S5: The results of enrichment of miRNA-199b targets.

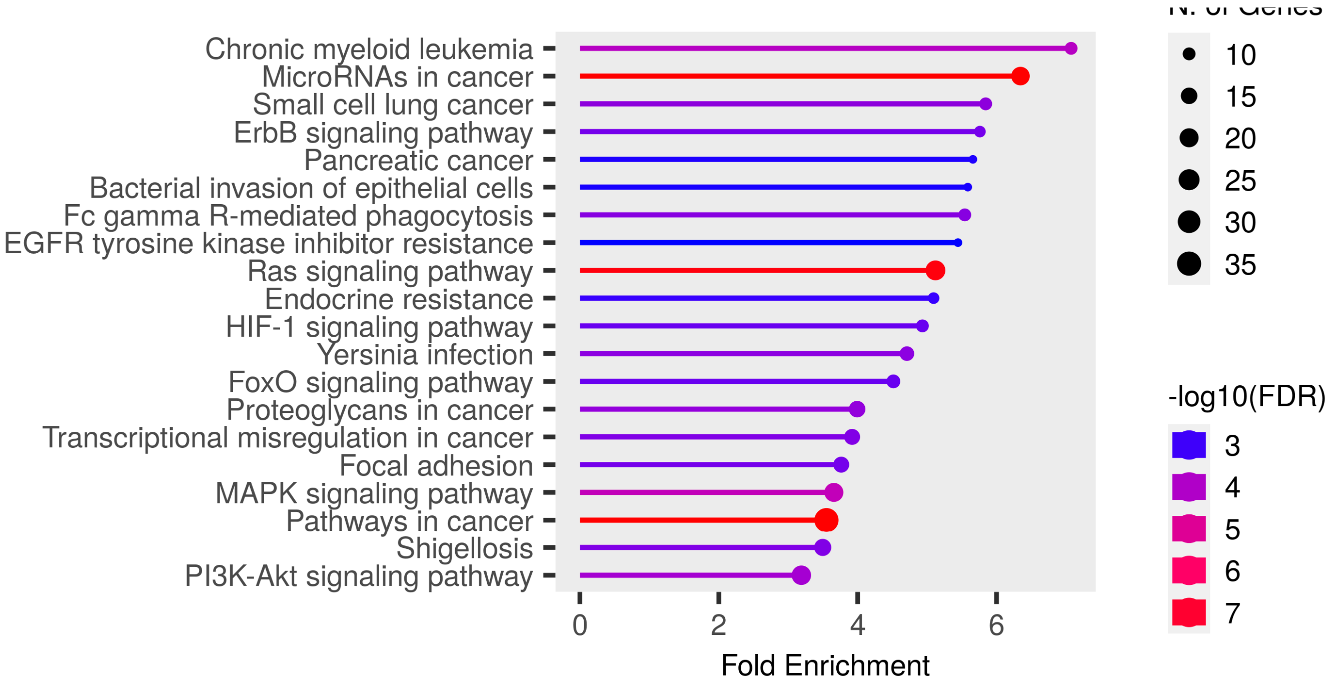

Figure S6: The results of enrichment of miRNA-200c targets.

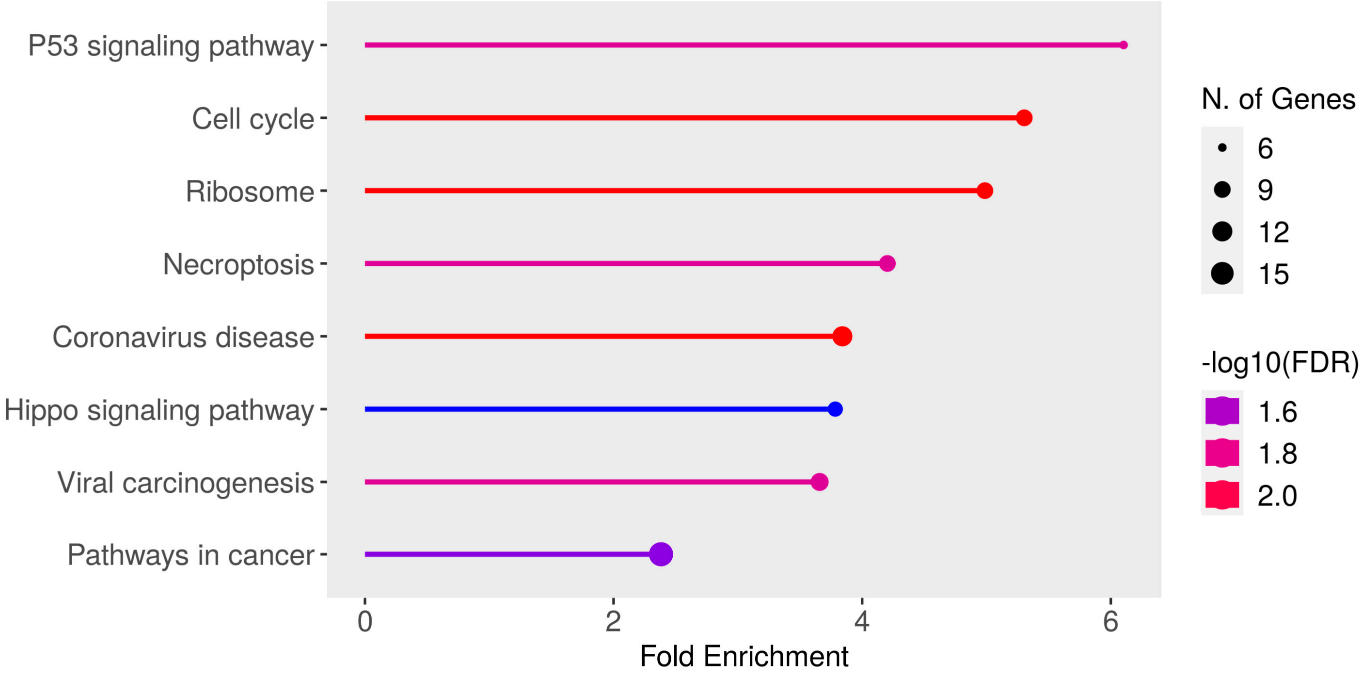

Figure S7: The results of enrichment of miRNA-455 targets.

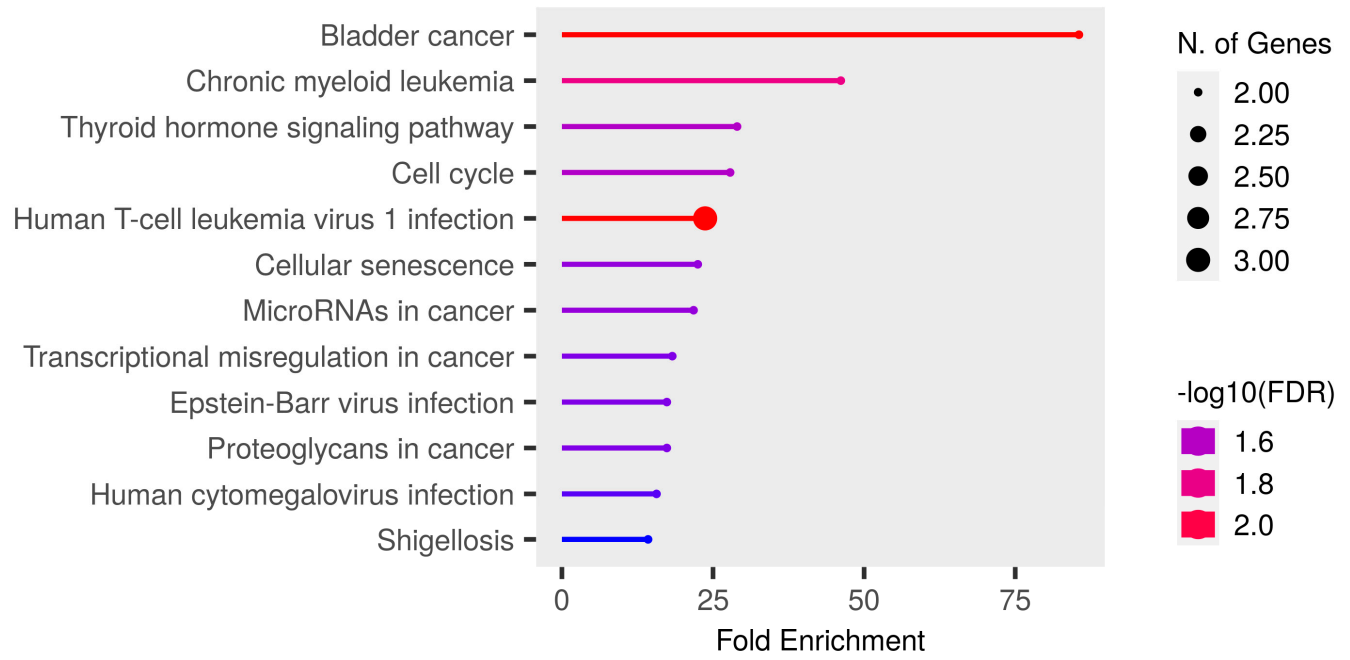

Figure S8: The results of enrichment of miRNA-487-3p targets.

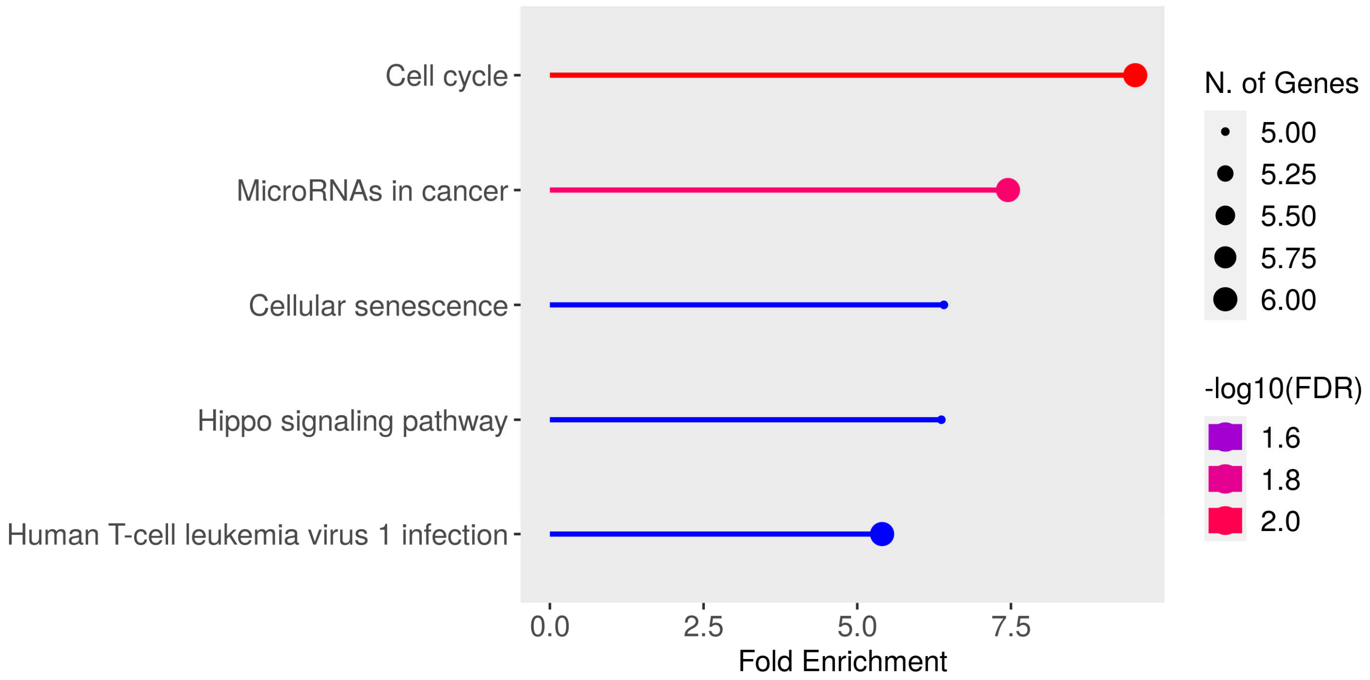

Figure S9: The results of enrichment of miRNA-582-3p targets
